# How does choice of residential community affect the social integration of rural migrants: insights from China

**DOI:** 10.1186/s40359-024-01617-9

**Published:** 2024-03-04

**Authors:** Qingjun Zhao, Guosong Wu, Hanrui Wang, Noshaba Aziz

**Affiliations:** 1 College of Economics and Management, Huzhou College, Huzhou, China; 2 School of Economics and Management, Huzhou University, Huzhou, China; 3 College of Economics and Management, Nanjing Agricultural University, Nanjing, China; 4 School of Economics, Shandong University of Technology, Zibo, China

**Keywords:** Social integration, Residential community choice, Residential space, Rural migrants, China, Power of space

## Abstract

The construction of public space is a new and important way to integrate rural migrants into urban society. Existing studies mainly discussed the factors affecting the social integration of rural migrants from the micro-individual and macro-system levels. Still, they seldom analyzed the differences between rural migrants’ residential communities and the roles these differences play in their social integration, especially from the perspective of residential space. Based on the data of the 2014 China Migrants Dynamic Monitoring Survey, this paper systematically examines the impact of residential community selection on the social integration of rural migrants and its possible effects using OLS, 2SLS, CMP, omitted variable test method, and KHB mediating effect model. It is found that the choice of residential community has a significant positive impact on the social integration of rural migrants, and the social integration of rural migrants living in formal communities has increased by 2.44%-3.20%. To overcome the potential endogeneity problems and selection bias of the empirical model, the study further adopted an instrumental variable estimation approach, combined with the omitted variable method for robustness check; the results still revealed the positive effect of living in formal communities on the social integration of rural migrants. The heterogeneous results showed that living in formal communities has a greater effect on the social integration of women and older-generation rural migrants. The farther the migration range and the longer the residence time of rural migrants, the greater the effect of living in the formal community on their social integration. Further mechanism testing revealed that living in formal communities not only directly enhances the social integration of rural migrants but also indirectly improves their social integration through public resource allocation, human capital accumulation, social status screening, and social network expansion. The indirect effect of capital accumulation is even greater. Therefore, to accelerate the full integration of rural migrants into urban society and achieve real urbanization and citizenship, the study proposes that the government should scientifically plan the layout of rural migrants’ living space and the construction of supporting facilities.

**Supplementary Information:**

The online version contains supplementary material available at 10.1186/s40359-024-01617-9.

## Introduction

Many rural migrants are constantly moving from rural areas to urban areas for work, and it has become one of China's main drivers of social change. However, due to China’s special dual economic structure system, rural migrants have long been on the fringes of cities, resulting in a “passing-through” mentality, making it difficult to grab a place in cities. Therefore, only by boosting rural migrants to break through psychological barriers, adapt to changes in social roles, and enhance their sense of belonging and identity in the city can rural migrants hope to achieve real citizenship. The construction of public space is a new and important way to achieve the integration of rural migrants and urban residents. In 2020, the Fourteenth Five-Year Plan for National Economic and Social Development of the People’s Republic of China and the Outline of the Vision for 2035 emphasized improving the policy of linking the scale of new urban construction land and the urbanization of agricultural transfer population. It proposed effectively increasing the supply of affordable housing, strengthening the guarantee of basic public services, and speeding up the urbanization of the agricultural transfer population. For rural migrants, housing is where they settle down in the city. Living in the city is the premise of rural migrants’ integration into the city. In accelerating the process of promoting the full integration of rural migrants into urban society, the issue of their living space cannot be ignored.

Existing studies have shown that most rural migrants choose to live in impoverished neighborhoods in urban centers or areas bordering urban and rural areas, with very poor living conditions [1–3]. Recent studies revealed that stable housing can promote the long-term settlement of rural migrants in cities and towns by improving the social integration of migrants [4, 5]. The closer the rural migrants live to the urban area and the closer they are to the living space of urban residents, the easier they are to form an urban identity [6]. Community, where rural migrants and locals reside next to each other and live together, is also the basis for rural migrants to gain a sense of home construct and their overall awareness and sense of spiritual belonging in the city [7]. So, do residential community choices affect the social integration of rural migrants? Further, if the choice of residential community affects the social integration of rural migrants, what is the mechanism of action? Will the effect of improving the living space environment on the sense of social integration present heterogeneous characteristics within the rural immigrant group? To answer these questions, the current paper offers detailed empirical evidence. The research conclusions of this paper can not only provide empirical evidence for government departments to formulate relevant policies and provide decision-making reference for promoting the urbanization process of rural migrants and achieving the new urbanization strategy.

Based on the policy mentioned above, background and research facts, this paper attempts to comprehensively evaluate the micro-influence, heterogeneous effect and transmission mechanism of the choice of residential community on the social integration of rural migrants from the perspective of residential space. Unlike the previous studies, the study's main contributions are as follows: Firstly, we attempt to focus on the living space status of rural migrants in the inflow area and the impact of residential community choice on the social integration of rural migrants. To a certain extent, our research on the social integration of rural migrants has been expanded. Second, starting from the mechanisms of public resource allocation, human capital accumulation, social status screening, and social network expansion implied by residential space, the KHB mediating effect model is used to identify the logical chain and possible mechanism by which residential community choice affects the social integration of rural migrants. The results deepen the relevant research on the social integration of rural migrants and provide theoretical reference and empirical evidence for promoting the construction of urban public space and accelerating the design of policies to accelerate the full integration of rural migrants into urban society.

The rest of this paper is structured as follows: The next section (2) reviews the literature on rural migrants’ choice of residential community and social integration and puts forward the research hypothesis. Section "Materials and Methods" introduces the data sources, primary variables, and estimation strategies. The empirical results regarding the overall effects, heterogeneous effects, and mechanisms of action of residential community choice on the social integration of rural migrants are presented and discussed in section "Empirical results and analysis". Section "Heterogeneity analysis and mechanism discussion" concludes the study with several policy implications. The study limitations are also presented in this section.

## Literature and hypotheses

### Literature review

The problem of social integration of rural migrants is complicated, and the solution to this problem is a long-term asymptotic process. Regarding the influencing factors of the social integration of rural migrants, various researchers have conducted in-depth analysis regarding the social integration of rural migrants from multiple disciplines and perspectives, such as economics, sociology, and psychology, and has achieved rich results. Existing studies have shown that rural migrants are subject to many constraints in urban integration [8, 9]. On the one hand, social and economic distances arise from the household registration system, social security, and wage levels. The current population floating and inability to settle in cities and towns is mainly caused by China’s institutional obstacles (especially the household registration system) [10, 11]. Most rural migrants are engaged in tiring and dangerous jobs [12], their income level is relatively low, and their awareness of social insurance participation is also poor. When they encounter greater risks (such as serious work-related accidents, serious diseases, etc.), then certain fluctuations arise in their normal life [13]. There is a huge gap between rural migrants and urban residents, especially in their concepts and knowledge level, which makes it difficult for them to integrate into urban society. Influenced by traditional thinking concepts and lifestyles, most rural migrants lack the economic willingness and ability to reinvest in improving their cultural quality and vocational skills [14, 15], resulting in the lack of employment competitiveness and difficulty in truly integrating into urban life. This also leads to a lack of security and a sense of belonging in the city, which makes it impossible for rural migrants to establish and develop in the city for a long time. In addition, the communication circle formed by kinship and geography is highly dependent on the traditional local network, which makes rural migrants lack the channels and opportunities to communicate with residents after entering the city, strengthening the rural migrants’ sub-regional relationship in the city. The social-ecological environment greatly reduces the urban identity and sense of belonging of rural migrants [16, 17].

In recent years, some studies have incorporated social mechanisms into the social framework for analyzing rural migrants and proposed that housing is an intermediate mechanism for social segmentation and integration [18, 19]]. The living area's characteristics significantly impact the attitudes and behaviors of rural migrants, and ownership of housing property has become an important indicator to measure individuals' or families' economic ability and achievement [20]. From the perspective of housing property rights and living quality, homeownership plays an important role in affecting migrants’ perceived social integration, existing studies generally agreed that local housing purchases promote the urban integration of rural migrants [21, 22]. The urbanization process has formed the transformation of urban and rural communities; factors such as the strategic drive of community culture, community residents’ health services, and community adult sports development are conducive to promoting the integration of migrants into cities [23, 24]. Migrant workers who live in better housing conditions and enjoy housing support are more willing to settle in urban areas[25]. From the perspective of residence distribution, the strong social connection between rural migrants and the community in the destination is likely to encourage them to choose permanent settlement [26], while residential isolation weakens the communication and interaction between rural migrants and local citizens, intensifies social distance, and inhibits the social integration of rural migrants. Harald’s research also found that the living patterns of minority migrants profoundly impact community integration. The more diverse the immigrant ethnic group is, the more frequent the group communication and the stronger the community cohesion and identity [27]. Migrants’ social capital, the cultural differences of immigrant communities, and the community’s acceptance of heterogeneous cultures are also likely to affect the personal well-being of migrants, thereby affecting the level of community integration of rural migrants [28].

It is apparent from the literature that many factors affect the social integration of rural migrants. Most of them believe that improving the living environment will help in promoting the social integration of rural migrants. However, few studies conducted in-depth research regarding rural migrants’ choice of residential community and social integration from the perspective of residential space. For rural migrants, improving the living space environment provides an important opportunity and motivation for this group to achieve urban social embedding and class leap. Housing is not only a material entity that satisfies the living property but also a symbolic and status consumption activity, which has become an important symbol of social status [29]. In addition, given the significant differences in rents between formal and informal housing, this may lead to differentiation of housing choices among rural migrants and affect their social integration. Therefore, with the help of large-scale representative micro-survey data, this study is particularly conceptualized to study the impact of residential community choice on the social integration of rural migrants and the underlying mechanism.

### Research hypotheses

The social integration of rural migrants is a cumulative process that requires gradual economic integration, cultural acceptance, behavioral adaptation, and finally, identity. According to the social embedded theory, economic action is a kind of social action; individual economic actions and consequences are embedded in a specific economic system, cultural environment, and social structure [30, 31]. The biggest difference between rural migrants and native residents is that they have dramatically transformed their living spaces. The residence of rural migrants in the city is not only their physical space to shelter from wind and rain but also their living space and social interaction space. This living and social interaction space often obtains various urban resources, accumulates human capital and involves social interaction. Social capital, in turn, provides opportunities for them to integrate into mainstream urban society. The location, environment and other material resources contained in the living space, as well as the relationship network formed by the interaction between people, may have a certain impact on the social integration of rural migrants [32].

On the one hand, the differentiation of living space is accompanied by the difference in the spatial distribution of public service resources and social resources. The living structure also determines the social interaction space of individuals. On the other hand, as a spatial field where social interaction and human capital spillovers occur intensively, residential communities are more likely to achieve the dissemination and diffusion of various types of information such as welfare qualifications, employment, entrepreneurship, mutual aid and lending. Therefore, living in a formal community means having richer resources, such as public facilities and services. At the same time, rural migrants and urban residents have frequent daily social interactions due to proximity, enhancing their psychological identity and sense of belonging to the city. This will help rural migrants fully integrate into urban society.

In addition, the living space of rural migrants has multiple attributes of physical, economic, and social space. It is not only the geographical space for their living but also the spatial field where the acquisition of public resources and knowledge spillover occurs intensively. It is also a social interaction space formed by residential activities. On the one hand, different residential locations mean different access to resources and possibly different access to development opportunities and public services [33, 34]. The spatial structure of most Chinese cities is concentric circles. In other words, the closer the residential location is to the city center, the richer the public facilities and resources, and the more job opportunities available. If rural migrants live in formal communities are more likely to receive positive externalities from urban public resources.

**Fig. 1:**
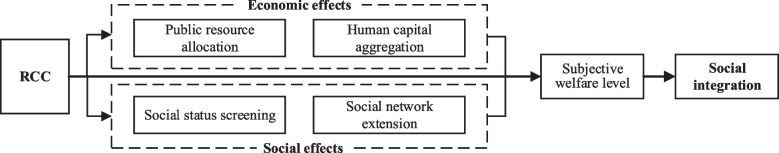
The impact path of RCC on social integration of rural migrants.

The function of human capital accumulation can also help them obtain better employment information employment opportunities and improve the level of human capital [35], thereby enhancing rural migrants’ sense of identity and belonging to the city where they are located, increasing the social integration of rural migrants. On the other hand, under the conditions of marketization, residence, as a symbolic and status-based consumption activity, is gradually alienated into a screening mechanism for social status [36, 37]. Rural migrants living in formal communities can increase their communication opportunities with urban residents. Imitation and learning will change the adaptation and acceptance of rural migrants to urban life, and it is easy to form psychological advantages. The psychological distance between rural migrants and urban and urban residents is shrinking, which improves their subjective status recognition and perception of respect and thus makes it easier for them to integrate into urban society. In addition, living in a formal community can also increase the opportunities for rural migrants to contact residents, optimize their social network structure, eliminate urban residents’ prejudice and discrimination against rural migrants, and meet rural migrants’ psychological needs and needs for emotional communication. This helps rural migrants integrate into urban society [38]. In short, differences in residential communities will not only affect the acquisition of public resources and the accumulation of human capital for rural migrants but also cause the differentiation of rural migrants’ subjective cognition and the reconstruction of social networks. These changes lead to differences in the social integration of rural migrants. According to the above analysis, this paper constructs the following framework (shown in Fig. 1) and proposes the following research hypotheses:

Hypothesis 1: Living in formal communities contributes to the social integration of rural migrants.

Hypothesis 2: The residential community choice (“RCC”) not only directly affects the social integration of rural migrants but also enhances their social integration through mechanisms such as public resource allocation, human capital accumulation, social status screening and social network expansion.

## Materials and methods

### Data

The micro-data used in this study comes from the 2014 China Migrants Dynamic Survey (“CMDS“), a recent survey conducted by the National Health and Family Planning Commission of China. The sample covers eight pilot cities of social integration, including Beijing, Qingdao, Xiamen, Jiaxing, Shenzhen, Zhongshan, Zhengzhou and Chengdu. The above cities are generally distributed in the eastern, central and western regions of China, and they are also the inflow places where the floating population is relatively concentrated and representative. The survey uses the Proportional Population Size sampling method to select the inflowing population aged 15 and above who have lived in a community for over a month and do not hold county registration. It has professional, scientific and large sample characteristics and can comprehensively describe the current situation of social integration of rural migrants in China. The total number of CMDS 2014 data samples is 16,000. Since this paper focuses on rural migrants working and doing business in cities and towns, 13,134 valid samples are screened out after excluding samples with missing key variables.

### Variables

#### Explained variable

The explained variable is social integration. Social integration is a process of cooperation and adaptation among different individuals, groups or cultures [39], which involves four aspects of integration: economy, society, culture and psychology. The economic aspect is the foundation, while psychological integration is the key to social integration [14, 40]. Psychological integration is crucial for social integration. Only when rural migrants genuinely integrate psychologically into the urban society of the destination can their goals of social integration be truly achieved. Therefore, this paper focuses on the psychological aspect of social integration among rural migrants and, considering data availability, measures their social integration status based on their perceptions and experiences of the urban living environment. Factor analysis was carried out based on the scores of several items of the respondents’ urban feelings in the questionnaire. The questionnaire contains four items: “I feel that I belong to this city,” “I feel that I am a member of this city,” “I see myself as part of this city,” “I would like to integrate into the community and be a member of it,”. It is worth noting that we chose these questions with the aim of capturing various aspects of psychological integration, ensuring a comprehensive exploration of the relationship between rural migrants and the city. Although there are subtle differences in wording, these variances may be intended to capture multiple dimensions of psychological integration, allowing for a more comprehensive understanding of migrant workers' psychological identification with the city. This design considers the complexity of psychological research, better elucidating the subtle relationship between individuals and the city. Specifically, Question 1 emphasizes the sense of belonging of rural migrants to the city, involving the emotional connection between individuals and the city; Question 2 emphasizes the identity recognition of rural migrants as members of the city, involving the individual's sense of participation and responsibility towards the city; Question 3 evaluates the overall attitude of rural migrants towards the integration with the city; These three questions form a progressive relationship, comprehensively examining the psychological identification of rural migrants with the city, starting from their sense of belonging, moving on to identity recognition, and ultimately exploring overall integration. Question 4 emphasizes the willingness of rural migrants to integrate into urban communities. The corresponding options include completely disagree, disagree, basically agree, and completely agree, with the values of 1, 2, 3, and 4. The higher the score, the higher the social integration of the respondent. We performed exploratory factor analysis on the scores of the above four items; the KMO value was 0.807, and the *P* value of the Bartlett sphericity test was 0.000, indicating that the factor analysis method was appropriate. Taking the eigenvalue greater than 1 as the standard to retain one factor (the cumulative variance explanation rate reaches 75.26%), this paper uses the comprehensive factor score of this factor to measure the social integration of rural migrants.

#### Explanatory variable

The explanatory variable is the choice of the residential community. Referring to the existing studies [41, 42], the choice of the residential community of rural migrants is measured by the item “What kind of community do you currently live in” in the CMDS2014 questionnaire. “Community,” “community of government agencies and institutions,” and “community of industrial and mining enterprises” are classified as formal communities, and “unrenovated old urban areas,” “urban villages or shanty towns,” “suburban fringes” and “rural communities” are classified as informal communities, respectively. The assigned values are 1 and 0. Within the sample range, 10,065 rural migrants live in formal communities, accounting for 76.63%.

#### Instrumental variable

Existing literature generally used village-level indicators as instrumental variables for individual-level indicators [42, 43]. Therefore, in this paper, the proportion of other migrants living in formal communities in the community where rural migrants live is used as an instrumental variable to correct the estimation bias caused by endogeneity. According to the CMDS2014 questionnaire data, this paper takes the communities where rural migrants live and their household registration places as grouping variables, divides them into several groups, calculates the proportion of other migrants living in formal communities in their groups and uses them as instrument variables to estimate. Generally speaking, the choice of a residential community for rural migrants is likely affected by the proportion of other migrants in the group (especially those in the same household registration place) living in formal communities. The larger the proportion of other migrants living in the formal community, the larger the probability of rural migrants choosing to live there. However, this proportion is not likely to directly affect the social integration of rural migrants.

#### Mediating variables

This paper regards public resource allocation, human capital accumulation, social status screening and social network expansion as the mechanisms by which rural migrants’ choice of community affects their social integration. Among them, public resources are measured by the questionnaire item “Have you established a resident health file in the community where you live?” The assigned values are 1 and 0. Within the sample, 3,093 rural migrants have established health records, accounting for 23.55%—29.23% of the rural migrants living in formal communities having health records. According to the method proposed by Barro and Lee [44], this study uses the average years of education of all individuals in the communities where rural migrants live as an indicator to measure human capital accumulation. Social status is measured by the questionnaire item “Where are you respected compared to people in the whole society?” and respondents answer the options on a scale of 1-10, representing 10 levels from low to high, 1 represents the lowest status in the society, and 10 represents the highest status in the society. Within the sample range, the average social status of rural migrants is 5.1129. Among them, the average social status of rural migrants living in formal communities is 5.2199. The questionnaire item also measures the social network “Are you currently a member of a trade union, volunteer association, fellow villagers’ association, etc.” assigning a value of 1 if you have participated in one or more of these organizations and assigning a value of 0 if you have not participated in any of them. Within the sample range, the mean value of a rural immigrant social network is 0.2617. Among them, the mean value of the social network of rural migrants living in formal communities is 0.3112.

#### Control variables

Based on the data of CMDS 2014 and the practice of existing literature, this paper controls various potential confounding factors that may affect the choice of a rural immigrant living community and social integration at the same time, mainly including: age, sex, education level, marital status, health status, income level, range of mobility, local residence time, residential property rights, employment status and other basic personal characteristics. The meaning of each variable and its descriptive statistics are shown in Table 1.

**Table 1.** Definitions and descriptions of variables

**Variables**	**Definition**	**Mean**	**Std.**
Social integration	Composite factor scores obtained by factor analysis methods	0.7471	0.1898
RCC	1=Formal community, 0=Informal community	0.2337	0.4232
Age	Respondent's age in 2014 (years)	31.9980	8.7502
Age squared	Age*Age/100	11.0043	5.9820
Gender	1=Male, 0=Female	0.5668	0.4955
Education	1=High school and above, 0=Below high school	0.3454	0.4755
Marriage	1=Married, 0=Unmarried	0.7269	0.4456
Health	Self-assessment of health status	3.7644	0.9692
Income	The logarithm of average monthly household income (Yuan)	8.5170	0.5886
Inter-provincial flows	1=Yes, 0=No	0.0356	0.1852
Inter-city flows	1=Yes, 0=No	0.4292	0.4950
Inter-county flows	1=Yes, 0=No	0.5353	0.4988
Length	Duration of residence in the city (years)	3.7210	4.3779
Residence ownership	1=Yes, 0=No	0.0739	0.2615
Employer	1=Yes, 0=No	0.2941	0.4557
Instrument variable	Proportion of other migrants of the same household origin living in formal communities in the communities where rural migrants are located	0.2362	0.3832
Public resources	Do you have a health record in your local community? 1=Yes, 0=No	0.2355	0.4243
Human capital	Average years of education of all individuals in the community (years)	10.0528	1.2408
Social status	Where do you stand in terms of respect compared to people in society as a whole? 1-10 points, the lowest is 1 point, the highest is 10 points	5.1129	1.7359
Social networks	Do you currently participate in local social organizations? 1=Yes, 0=No	0.2617	0.4396

### Empirical strategies

First, this paper constructs the following regression equation to evaluate the impact of residential community choice on the social integration of rural migrants:

1 $${Integration}_{i} ={a}_{0}+{a}_{1}{RCC}_{i}+{a}_{2}{W}_{i}+{a}_{3}{City}_{i}+{\varepsilon }_{i}$$

In eq. (1), ${Integration}_{i}$ represents the social integration of the ${i}_{th}$ rural migrant, ${RCC}_{i}$ represents the choice of the ${i}_{th}$ rural migrant’s residential community; ${W}_{i}$ is the control variable, and ${City}_{i}$ represents the urban dummy variable; ${a}_{j}(j=0, 1,\mathrm{ 2,3})$ is the parameter to be estimated in the equation, and ${\varepsilon }_{i}$ is the random disturbance term.

Second, it should be noted that there may be an endogeneity problem between rural migrants’ choice of living community and social integration. On the one hand, the residential community selection of rural migrants may result from self-selection, and the residential community selection variable may not satisfy random sampling. Directly regression with it may cause selection bias in the estimation results due to non-random sampling. On the other hand, the factors affecting the social integration of rural migrants are complex, and it is difficult to control them completely in the model. Other unobservable factors may affect the choice of rural migrants’ residential community and social integration simultaneously; that is, the problem of missing variables. The instrumental variable method is a conventional means to solve the endogeneity problem, which requires constructing a regression equation of residential community selection and its instrumental variables before eq. (1) is estimated:

2 $${RCC}_{i}={b}_{0}+{b}_{1}{Z}_{i}+{b}_{2}{W}_{i}+{b}_{3}{City}_{i}+{u}_{i}$$

In eq. (2), ${Z}_{i}$ is the instrumental variable, ${b}_{j}(j=0, 1,\mathrm{ 2,3})$ is the parameter to be estimated, and ${u}_{i}$ is the error term. Then, this paper adopts the 2SLS model to correct the possible endogenous bias of the underlying regression process. At the same time, considering that the potential endogenous variable residential community selection is a binary variable, the 2SLS model ignores the categorical variable attributes of residential community selection to a certain extent and cannot fully utilize the information, resulting in a loss of estimation efficiency. To this end, this paper introduces the Conditional Mixed Process (CMP) method, which can fit a series of multi-equation, multi-level and conditional recursive mixed process estimators and re-estimate the instrumental variables within a unified CMP framework [45]. Based on seemingly uncorrelated regression, CMP constructs a system of recursive equations based on the maximum likelihood estimation method, which requires simultaneous estimation of eqs. (1) and (2). The value of the correlation coefficient of the error terms of the two equations can be used to judge whether the residential community selection variable is endogenous. If the value significantly differs from 0, the CMP estimation result is better than the benchmark regression result [45].

Third, we also solve the endogeneity problem caused by missing variables; this paper uses the method proposed by Oster to test the potential missing variables and their impact on the regression results [46]. When there are some unobservable missing variables in the regression model, it can be obtained by calculating the estimator ${\upbeta }^{*}$ approximately consensus estimates of residential community choice on social integration of rural migrants:

3 $${\upbeta }^{*}\approx \widetilde{\upbeta }-\updelta \left({\upbeta }^{0}-\widetilde{\upbeta }\right)\times \left({R}_{{\text{max}}}-\widetilde{R}\right)/\left(\widetilde{R}-{R}^{0}\right)$$

In eq. (3), ${\upbeta }^{*}$ represents the impact of residential community selection on the social integration of rural migrants, ${\upbeta }^{0}$ and ${R}^{0}$ represent the parameter estimates and goodness of fit of residential community selection when constrained control variables are added. $\widetilde{\mathrm{\rm B}}$ and $\widetilde{R}$ represent the parameter estimates and goodness of fit selected by the residential community when all observable variables are added as control variables, respectively. $\updelta$ represents the ratio of observable and unobservable variables to the explanatory power of rural immigrant social integration. ${R}_{{\text{max}}}$ represents the maximum goodness of fit of the regression equation when all omitted variables can be included in the model. According to Oster’s suggestion [46], we adopt the following identification strategy to test the effect of omitted variables, assuming that ${R}_{{\text{max}}}$ is 1.3 times, 1.4 times, 1.5 times and 1.6 times the goodness of fit of the current regression equation, and when $\beta$=0, if $\updelta$ The value is greater than 1, indicating that the omitted variable will not change the influence of the explanatory variable on the explained variable.

Fourth, to further verify the mechanism of the four aspects of public resource allocation, human capital accumulation, social status screening and social network expansion in the impact of residential community choice on the social integration of rural migrants, we use the mediating effect proposed by Baron and Kenny [47], in addition to eq. (1), it is necessary to construct the following regression equation:

4 $${M}_{i}={c}_{0}+{c}_{1}{RCC}_{i}+{c}_{2}{s}_{i}+{\varepsilon }_{i}$$

5 $${Integration}_{i}={d}_{0}+{d}_{1}{RCC}_{i}+{d}_{2}{M}_{i}+{d}_{3}{s}_{i}+{\varepsilon }_{i}$$

In eqs. (4) and (5), ${M}_{i}$ represents mediating variables, including public resources, human capital, social status, and social networks. On the basis that eq. (1) has confirmed that the choice of the residential community significantly affects the social integration of rural migrants if both ${c}_{1}$ and ${d}_{2}$ are significant, there is an indirect effect. At this time, when ${d}_{1}$ is not significant, there is a complete mediation effect. When ${d}_{1}$ is significant and ${d}_{1}$<${a}_{1}$, there is a partial mediation effect. At the same time, this paper further adopts the KHB method proposed by Karlson et al. [48] to decompose and statistically test the effect of the choice of residential community on the social integration of rural migrants through mediating variables.

## Empirical results and analysis

### Baseline regression results

Table 2 reports the benchmark regression results of the impact of residential community choice on the social integration of rural migrants. We adopt a stepwise regression method to verify the regression results' robustness. Column (1) only controls the core explanatory variables; the city dummy variable is added to the (2) column, the control variables are added to the (3) column, and the control variables and city dummy variables are added to the (4) column. It can be seen from Table 2 that whether only the core explanatory variables are controlled or control variables and urban dummy variables are added, the direction and significance of the impact of residential community choice on the social integration of rural migrants are consistent, indicating that the estimation results are very robust. It can be seen from the results in column (4) that living in informal communities will significantly increase the social integration of rural migrants by 2.44% compared to rural migrants living in informal communities. The above results show that living in formal communities helps promote the social integration of rural migrants, and hypothesis 1 is preliminarily confirmed.

**Table 2:** OLS model results of factors determining social integration

**Variables**	**(1)**	**(2)**	**(3)**	**(4)**
RCC	0.0514*** (0.0039)	0.0400*** (0.0039)	0.0267*** (0.0040)	0.0244*** (0.0040)
Age			-0.0001 (0.0015)	-0.0011 (0.0015)
Age squared			0.0008 (0.0021)	0.0017 (0.0021)
Gender			-0.0062* (0.0032)	-0.0065** (0.0032)
Education			0.0073** (0.0035)	0.0087** (0.0036)
Marriage			0.0101* (0.0053)	0.0125** (0.0054)
Health			0.0263*** (0.0018)	0.0274*** (0.0018)
Income			0.0022 (0.0033)	0.0098*** (0.0033)
Inter-city flows			-0.0535*** (0.0079)	-0.0411*** (0.0080)
Inter-provincial flows			-0.1127*** (0.0080)	-0.0559*** (0.0088)
Length			0.0018*** (0.0004)	0.0019*** (0.0004)
Housing			0.0620*** (0.0064)	0.0595*** (0.0064)
Employer			0.0089** (0.0038)	0.0048 (0.0038)
City effects		YES		YES
Constant	0.7351*** (0.0019)	0.6883*** (0.0046)	0.6805*** (0.0350)	0.5551*** (0.0355)
R-squared	0.0132	0.0678	0.0752	0.1037
Observations	13,134	13,134	13,134	13,134

Robust standard errors in parentheses. *** *p* < 0.01, ** *p* < 0.05, * *p* < 0.1

In addition, from the estimation results of the control variables in column (4) of Table 2, the degree of social integration of female rural migrants is significantly higher than that of males, which is consistent with the conclusion of most literature. It is mainly because male rural migrants are more stressed than females living in cities, resulting in less social integration than females [6, 49]. Education level, marital status, health status and income level significantly and positively impact the social integration of rural migrants, indicating that rural migrants with higher education, married, healthy and higher income levels have higher social integration. Relevant studies have also found similar results. Rural migrants with higher education levels have relatively higher employment and income levels in urban areas, and their economic ability to integrate into cities is also higher [50]. Because the married rural migrants have stable families, their main purpose of living in the city is to maintain family life, so the social integration degree is higher than that of the unmarried rural migrants. Healthy human capital is an important factor for rural migrants to realize the transition from low-level integration to high-level integration [51]. Good health helps to enrich the urban life of rural migrants [52], and their social integration will naturally be higher. Inter-urban and inter-provincial rural migrants have significantly lower levels of urban integration than rural migrants who move within the same city. The farther the migration distance is, the lower the degree of social integration. This may be due to the large differences in language communication, living habits and cultural traditions between the inflowing city and the place of hukou, which increases the difficulty of urban integration of rural migrants [53]. The coefficient of migration time is significantly positive, indicating that the longer the rural migrants stay in the inflow area, the higher the degree of social integration. It also reflects the positive impact of the stability of rural migrants’ work and life on psychological integration. At the same time, the longer the rural migrants stay in the inflow area, the closer the social distance between them and the locals, the more they want to become citizens [54]. Compared with rural migrants without housing property rights, rural migrants with housing property rights have a higher degree of social integration, consistent with previous research by scholars. Housing property rights have become an important indicator to measure individuals' or families' economic ability and achievement [10, 22, 41].

### Endogeneity

#### Instrumental variable checks

Although this paper controls the variables that may impact the choice of residential community and social integration of rural migrants in the benchmark regression analysis, the empirical analysis may still have potential endogeneity problems such as self-selection bias and omitted variable bias. We further analyzed the influencing factors of rural migrants' residential community selection. Table A1 reports the estimation results of the Probit model. The estimates indicate that male, higher education, higher income, and rural migrants with property ownership are more likely to choose formal communities. In contrast, married individuals, those who have migrated across provinces, and those who are self-employed are less likely to choose formal communities. This suggests that the residential community selection of rural migrants is not a random assignment. To this end, we use the 2SLS model for further analysis, and Table 3 reports the estimation results of the 2SLS model. From the estimation results of the first stage of the 2SLS model in column (1), the impact of instrumental variables on the choice of residential community is significantly positive at the 1% statistical level, which means that the instrumental variables meet the correlation conditions. At the same time, the Cragg-Donald Wald F statistic in the first stage is significantly greater than 10. It is significant at the 1% statistical level, indicating that the instrumental variables we selected strongly correlate with the core explanatory variables. The endogenous variables are just right in the case of identification without a weak instrumental variable problem. From the estimation results of the second stage of the 2SLS model in column (2), the heteroscedasticity robust Durbin-Wu-Hausman test value of residential community selection rejects the null hypothesis at the 1% significance level, indicating that residential community selection is indeed for endogenous variables, the estimation results of the 2SLS model are more reliable than the OLS model. From the second-stage regression results of the 2SLS model in column (2), after correcting possible endogenous biases, living in informal communities will significantly improve the social integration of rural migrants. The estimated coefficient of the 2SLS model is 0.0307, ​​the coefficient in column (4) of Table 2 becomes larger, and the standard error is also larger, indicating that the benchmark regression underestimates the effect of residential community choice on the social integration of rural migrants.

**Table 3:** The effects of RCC on social integration: IV model test results

**Variables**	**2SLS**		CMP	
	**(1)**	**(2)**	**(3)**	**(4)**
RCC		0.0307***		0.0320***
		(0.0052)		(0.0047)
Instrument variable	0.9041*** (0.0068)		0.9040*** (0.0068)	
Cragg-Donald Wald F	23574.0000***			
Durbin-Wu-Hausman F		7.9753***		
atanhrho_12				-0.0342***
Control variables	YES	YES	YES	YES
City effects	YES	YES	YES	YES
Constant	-0.1291** (0.0514)	0.5748*** (0.0388)	-0.1283** (0.0514)	0.5554*** (0.0355)
R-squared	0.7150	0.1057		
Observations	11,309	11,309	11,309	11,309

Robust standard errors in parentheses. *** *p* < 0.01, ** *p* < 0.05

In addition, since the endogenous variable, residential community selection, is a binary variable, using the 2SLS model reduces the validity of the estimated results to a certain extent. To solve this problem, this paper further utilizes the CMP method for estimation. From the regression results of the first stage of the CMP method in column (3), it can be seen that the instrumental variables have a significant positive impact on the choice of residential community, which also verifies that the instrumental variables meet the correlation requirements. The second-stage result in column (4) shows that the choice of residential community has a significant positive impact on the social integration of rural migrants, with a coefficient of 0.0320, which is larger than the coefficient in column (2), indicating that the 2SLS model estimates have a loss of validity problem. The endogeneity test parameter of the CMP method is significant at the 1% statistical level, rejecting the null hypothesis that the choice of residential community is an exogenous variable, indicating that the instrumental variable method is reasonable. After endogenous treatment, the choice of residential community has a significant positive impact on the social integration of rural migrants, and this result is highly consistent with the estimation results of the 2SLS model and the OLS model. The positive impact of residential community choice on the social integration of rural migrants is robust, and research hypothesis 1 is further confirmed.

#### Omitted variables checks

In addition to the endogeneity problem of self-selection bias, this paper may also have the problem of missing variables. Although we controlled for many individual and city-level characteristics variables in our empirical model, there were still the omission effects of unobservable variables for which data were not collected. To solve this problem, we examine potential omitted variables and their impact on the regression process according to the method proposed by Oster [46]. As shown in Table 4, when β=0, regardless of whether ${{\varvec{R}}}_{max}$ is 1.3 times, 1.4 times, or 1.5 times, 1.6 times, all δ is greater than 1, and the test is passed. It shows that the coefficient of the choice of residential community on the social integration of rural migrants is relatively stable. Therefore, we can infer that even if there are missing variables, the judgment of this paper on the relationship between the choice of residential community and social integration of rural migrants is still robust; that is, living in a formal community can significantly improve the social integration of rural migrants, which once again confirms the research hypothesis 1.

**Table 4:** Omitted Variables Checks results

**Variables**	**Standard of judgment**	${{\varvec{R}}}_{max}=1.3\widetilde{{\varvec{R}}}$	${{\varvec{R}}}_{max}=1.4\widetilde{{\varvec{R}}}$	${{\varvec{R}}}_{max}=1.5\widetilde{{\varvec{R}}}$	${{\varvec{R}}}_{max}=1.6\widetilde{{\varvec{R}}}$	**Pass the test**
RCC	$\delta$>1	2.1166	1.6124	1.3069	1.0987	YES

### Robustness

To further verify the reliability of the empirical results, we also conduct robustness tests by adjusting variables and samples. The results are shown in Table 5. First, we replaced the explanatory variables directly with the four measures of social inclusion, namely “I feel like I belong to this city,” “I feel like I am a member of this city,” “I see myself as a part of this city” “I would like to integrate into the community, be a part of it,” and then make a comeback. The empirical results of adjusting the explained variables are shown in columns (1)-(4), and the results show that even if the different measures of social integration are replaced, the conclusions in Table 2 are still drawn. Second, we also adjusted the sample (retaining only the 20-50-year-old sample) for re-regression, and the results are shown in column (5). The estimation results show that the choice of residential community is still very significant, and the coefficient is positive, indicating that living in a formal community still significantly improves the social integration of rural migrants, which further confirms the robustness of the core conclusion.

**Table 5:** The effects of RCC on social integration: Robustness test results.

**Variables**	**(1)**	**(2)**	**(3)**	**(4)**	**(5)**
RCC	0.0886*** (0.0145)	0.0837*** (0.0144)	0.0762*** (0.0137)	0.0448*** (0.0126)	0.0144*** (0.0023)
Control variables	YES	YES	YES	YES	YES
City effects	YES	YES	YES	YES	YES
Constant	2.2880*** (0.1354)	2.6369*** (0.1292)	2.7581*** (0.1216)	2.9483*** (0.1114)	0.5211*** (0.0459)
R-squared	0.0418	0.0413	0.0410	0.0412	
Observations	13,134	13,134	13,134	13,134	12,141

Robust standard errors in parentheses. *** *p* < 0.01

## Heterogeneity analysis and mechanism discussion

### Heterogeneity analysis

It has been concluded above that living in formal communities helps promote the social integration of rural migrants. However, it is worth noting that this is only the average effect of the whole sample level, without considering the internal differences and differentiation of rural migrants. To obtain more detailed research conclusions, we will further explore the heterogeneity of the impact of residential community choice on the social integration of rural migrants by grouping by gender, age, range of mobility, and length of stay.

**Fig. 2:**
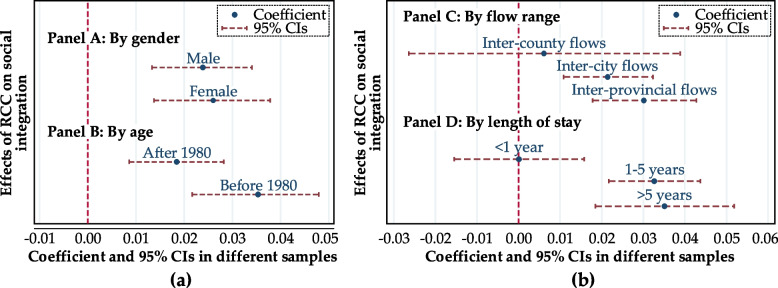
The heterogeneity effects of RCC on social integration. (Note: The control variables introduced in the model fit were consistent with those in Table 1.)

First, as more and more farmers leave the land and enter the cities, the female migrant population has gradually become an important part of the migrant population. Within the sample are 5,690 female rural migrants, accounting for 43.32%, and 7,444 male rural migrants, accounting for 56.68%. Panel A in Fig. 2(a) shows gender differences in the impact of residential community choices on the social integration of rural migrants. The results show that overall, for both male and female rural migrants, living in a formal community has a statistically significant positive effect of 1% on their social integration. However, there are differences in the impact effects. Compared with male rural migrants, living in formal communities has a greater impact on the social integration of female rural migrants. The difference is 0.21 percentage points. The possible explanation for this result is that, under the influence of traditional Chinese culture, female rural migrants pursue a stable housing security and living environment more than male rural migrants [55]. They are more sensitive to improving living space and live in formal communities. The impact on their social integration is also greater.

Second, intergenerational differences have always been the focus of research in the field of urbanization of the floating population [56]. The existing research generally divides the floating population into two groups, the new generation and the old generation, based on those born in 1980 [57], and there is obvious group heterogeneity between the two generations of rural migrants [58]. Therefore, by convention, we classify the rural migrants born before 1980 as the older generation of rural migrants and the rural migrants born in 1980 and later as the new generation of rural migrants. Within the sample are 8064 new-generation rural migrants, accounting for 61.4%, and 5,070 older-generation rural migrants, accounting for 38.6%. Panel B in Fig. 2(a) shows the generational differences in the impact of residential community choices on the social integration of rural migrants. The results show that, overall, for both the new and the older generation of rural migrants, living in formal communities significantly and positively affects their social integration at the statistical level of 1%. In terms of the impact effect, compared with the new generation of rural migrants, the choice of residential community has a greater impact on the social integration of the older generation of rural migrants. The difference is 1.64 percentage points. The reason for this result may be that the new generation of rural migrants has more ways and channels to integrate into the city than the older generation, and their urban integration is less affected by the interaction of their neighbors [56, 59], while the new generation of rural migrants has more channels to integrate into the city than the older generation. The older generation of rural migrants mainly through traditional social interaction, in addition to colleagues at work, the neighborhood interaction in the place of residence is their main choice. Therefore, the older generation of rural migrants may be more sensitive to the choice of living community and have a greater impact on their urban social integration process.

Thirdly, this paper divides the sample into three sub-samples: inter-provincial flow, intra-provincial inter-city, and intra-city inter-county sub-samples, and carries out regression analysis, respectively. Panel C in Fig. 2(b) shows the difference in the mobility range of the impact of residential community choice on the social integration of rural migrants. The results show that living in formal communities significantly improves the social integration of rural migrants who are inter-provincial and inter-city migrants, but living in formal communities has no significant impact on the social integration of rural migrants who are migrants across counties within the city. It can be seen that the farther the flow range is, the larger the administrative area it spans and the greater the effect of living space on the social integration of rural migrants. Among them, the inter-provincial flow is 0.87 percentage points higher than the inter-provincial flow. This is mainly because, with the expansion of the scope of movement, the uncertainty faced by rural migrants is also increasing. Improving living conditions and enhancing residential stability can draw in the social distance between rural migrants and urban residents, thereby reducing the social distance between rural migrants and urban residents. Promote their integration into urban society [60].

Fourthly, this paper divides rural migrants into three groups (less than 1 year, 1-5 years and more than 5 years) according to their residence time. The three groups are respectively subjected to regression analysis. Panel D in Fig. 2(b) shows the difference in residence time for the impact of residential community choice on the social integration of rural migrants. The results show that living in the formal community has a significant improvement effect on the social integration of rural migrants who have flown in for 1-5 years and more than 5 years, but living in the formal community has no significant impact on the social integration of rural migrants who have flown in for less than one year. It can be seen that the longer the rural migrants stay in the inflow area, the higher the probability of living in the formal community, and thus, the influence of the formal community on the rural migrants who have been inflowing for a long time becomes stronger. This is mainly because the longer the rural migrants stay in the inflow area, the stronger their adaptability to the city. Among them, the inflow of rural migrants for more than 5 years is 0.25 percentage points higher than the inflow of 1-5 years. They can establish a new social network relationship in the living community and have a stronger sense of belonging to the city psychologically. The degree of integration is also higher [61].

### Mechanism analysis: how does RCC affect social integration

Based on the previous theoretical analysis, this paper divides the impact of residential community choice on the social integration of rural migrants into two parts: economic and social effects. On this basis, the mediation effect model is used to analyze the distribution of public resources, human capital accumulation, social status screening and social status. Four aspects of social network expansion are used to empirically test the mechanism of residential community selection to enhance the social integration of rural migrants. The test results are shown in Table 6. Among them, columns (1)-(4) are listed as the test results of the indirect impact of the choice of residential community on the social integration of rural migrants through economic effects, and columns (5)-(8) show the test results of the indirect impact of the choice of residential community on the social integration of rural migrants through social effects.

**Table 6:** The effects of RCC on social integration: Mediating effects test results

**Variables**	**(1)**	**(2)**	**(3)**	**(4)**	**(5)**	**(6)**	**(7)**	**(8)**
RCC	0.0545*** (0.0093)	0.0236*** (0.0040)	0.5743*** (0.0221)	0.0217*** (0.0041)	0.0630* (0.0374)	0.0234*** (0.0040)	0.0337*** (0.0097)	0.0239*** (0.0040)
Public resources		0.0143*** (0.0039)						
Human capital				0.0046*** (0.0017)				
Social status						0.0151*** (0.0010)		
Social networks								0.0146*** (0.0037)
Total effect	0.0244*** (0.0040)		0.0244*** (0.0040)		0.0244*** (0.0040)		0.0244*** (0.0040)	
Direct effect	0.0236*** (0.0040)		0.0217*** (0.0041)		0.0234*** (0.0040)		0.0239*** (0.0040)	
Indirect effect	0.0008*** (0.0002)		0.0027*** (0.0010)		0.0009* (0.0006)		0.0005*** (0.0002)	
Control variables	YES	YES	YES	YES	YES	YES	YES	YES
City effects	YES	YES	YES	YES	YES	YES	YES	YES
Observations	13,134	13,134	13,134	13,134	13,134	13,134	13,134	13,134

Robust standard errors in parentheses. *** *p* < 0.01, * *p* < 0.1

First, the mediating effect of public resource allocation in column (1) shows that the choice of residential community significantly impacts public resources at the 1% statistical level, indicating that rural migrants living in formal communities are more likely to obtain public resources. Column (2) shows that residential community choice and public resources positively impact the social integration of rural migrants and are significant at the 1% statistical level. This paper further observes that the influence coefficient of residential community choice on the social integration of rural migrants has dropped from 0.0244 in column (4) of Table 2 to 0.0236. This indicates that the allocation of public resources plays a mediating role in the choice of residential community, affecting the social integration of rural migrants.

Second, the mediating effect of human capital accumulation in column (3) shows that the choice of residential community has a significant positive impact on human capital accumulation at the statistical level of 1%, indicating that rural migrants living in formal communities can help improve the degree of human capital accumulation. Further, column (4) shows that residential community choice and human capital accumulation positively impact the social integration of rural migrants and are significant at the 1% statistical level. At the same time, the influence coefficient of residential community choice on the social integration of rural migrants dropped from 0.0244 in column (4) of Table 2 to 0.0217. This indicates that human capital accumulation plays a mediating role in the choice of residential community, affecting the social integration of rural migrants.

Third, the mediating effect of social status screening in column (5) shows that the choice of residential community significantly positively impacts subjective social status at the 10% statistical level, indicating that rural migrants living in formal communities have a higher cognitive level of subjective social status. The results in column 6 show that residential community choice and subjective social status significantly positively impact the social integration of rural migrants. At the same time, the influence coefficient of residential community choice on the social integration of rural migrants dropped from 0.0244 in column (4) of Table 2 to 0.0234. This suggests that social status screening plays a mediating role in the process of residential community choice, affecting the social integration of rural migrants.

Fourth, the mediating effect of social network expansion in column (7) shows that the choice of living community has a significant positive impact on the social network at the 1% statistical level, indicating that living in a formal community helps to expand the social network of rural migrants. The results in column (8) show that the choice of a residential community and social network positively impacts the social integration of rural migrants at a 1% statistical level. At the same time, the influence coefficient of the choice of residential community on the social integration of rural migrants dropped from 0.0244 in column (4) of Table 2 to 0.0239. This suggests that social network expansion plays a mediating role in the process of residential community choice affecting rural migrants’ social integration.

In addition, we also decomposed the mediating effects of public resource allocation, human capital agglomeration, social status screening and social network expansion based on the KHB method. As shown in Table 6, the estimation results of the KHB method are highly similar to those obtained by the Baron and Kenny methods. The indirect effects of public resources, human capital, and social networks are all significant at the 1% level, the indirect effects of social status at the 10% level, and the coefficient signs are all positive. Further analysis shows that the indirect effects of public resources, human capital, social status and social network account for 3.28%, 11.07%, 3.69% and 2.05% of the total effects of residential community choice on the social integration of rural migrants. It can be seen that living in formal communities will not only directly enhance the social integration of rural migrants but also indirectly improve their social integration through public resource allocation, human capital accumulation, social status screening, and social network expansion. Among them, the indirect effect of human capital agglomeration is greater, more influence comes from the direct effect of the choice of residential community. This further confirms the robustness of the results of the mediation effect, and hypothesis 2 of this paper is confirmed.

## Conclusions and policy implications

### Conclusions

The residence of rural migrants in the city is not only their physical space to shelter from wind and rain but also their living space and social interaction space. This living and social interaction space often obtains various urban resources and accumulates human capital and social interaction for them. Social capital, in turn, provides opportunities for them to integrate into mainstream urban society. Housing is the place where rural migrants live in cities. In accelerating the process of promoting the full integration of rural migrants into urban society, the issue of their living space cannot be ignored. Regrettably, the existing research mainly focuses on the sense of belonging and subjective residence intention brought by housing, and there is a lack of in-depth quantitative research on the relationship between living space and rural migrants’ sense of social integration and subjective well-being. Very little literature has explored the potential impact of rural migrants’ housing policies and residential segregation on their life satisfaction. Still, the micro-influence of housing behavior on rural migrants’ social integration and its mechanism of action is usually ignored. Therefore, based on the CMDS2014 data, this paper systematically evaluates the impact of residential community selection on the social integration of rural migrants from the perspective of residential space, using the instrumental variable method and omitted variable test. Starting from the mechanisms of agglomeration, social status screening and social network expansion, and with the help of the mediation effect model, this paper deeply analyzes the possible mechanism by which the choice of residential community affects the social integration of rural migrants.

The research results show that living in formal communities enhances the social integration of rural migrants. This boosting effect still exists after using the 2SLS model and the CMP method to alleviate the potential endogeneity problem and multiple robustness tests such as omitted variable tests, replacement of explained variables, and adjustment of sample data. There is heterogeneity in the impact of the choice of residential community on the social integration of rural migrants. Compared with male and new-generation rural migrants, living in a formal community has a more significant role in improving the social integration of women and older-generation rural migrants. In addition, the farther the migration range and the longer the residence time of rural migrants, the greater the effect of living in formal communities on their social integration. The analysis of the action mechanism based on the mediation effect model shows that public resource allocation, human capital agglomeration, social status screening and social network expansion partially mediate between rural migrants’ choice of residential community and social integration. The KHB method is used to determine the mediation effect, and the effect is further decomposed. The results show that the impact of residential community choice on the social integration of rural migrants has a direct effect and indirectly improves the social integration of rural migrants through mechanisms such as public resource allocation, human capital agglomeration, social status screening, and social network expansion. The indirect effect of human capital agglomeration is even greater. At the same time, more influence comes from the direct effect of the choice of residential community.

### Policy implications

Based on the findings, the study proposes the following suggestions for policymakers. In promoting the urbanization of rural migrants, governments at all levels should focus on considering the key role of residential community factors for rural migrants to take root and integrate into cities.

First, pay attention to the impact of living space on rural migrants and fully understand the behavior and psychological adaptation of rural migrants in the decision-making process of residential communities. The construction of public space is a new and important way to realize the integration of rural migrants and urban residents. With the growth and development of the economy, there will be differences in the living standards and living quality needs of all social strata, and the differentiation of urban living space is an inevitable historical trend. Rural migrants should be encouraged and supported to live in formal communities, and the transformation of informal communities should be accelerated. As far as the design of commercial housing is concerned, more public space and shared facilities should be taken into account, which will provide the possibility of more contact and social interaction between rural migrants and citizens [42]. At the same time, housing renovation and community construction in urban village communities, shanty towns, and urban-rural fringes should also be accelerated.

Second, plan the residential space layout and fully use the residential location's economic and social resource advantages to enhance the social integration of rural migrants. For example, planning transportation infrastructure and public service facilities should consider the housing location and living conditions of rural immigrant groups. Avoid excessive concentration of resources in central locations, and arrange all aspects of supply facilities (water supply, power supply, gas supply, supply network, etc.) in some new formal communities and informal communities. Improve the surrounding public service facilities, such as business, education, medical care, entertainment, etc., to meet the reasonable living needs of rural migrants in different residential communities as much as possible. At the same time, in the process of urban transformation and renewal, attention should be paid to preserving the social relationships and networks of rural migrant communities. By creating a step-by-step and differentiated mixed living model, the living conditions of rural migrants are improved by classification, thereby reducing the negative impact of the differentiation of living spaces on the social integration of rural migrants.

Third, improve the subjective status cognition of rural migrants and optimize the social network structure of rural migrants from multiple perspectives to enhance the sense of urban belonging and social integration of rural migrants. Through strengthening the investment in vocational training and education, giving appropriate subsidies and preferential treatment, the income level of rural migrants should be improved, and the policy difference between urban and rural household registration should be gradually eliminated. The social status of rural migrants should be improved to improve their subjective status cognition and perception of respect [62]. At the same time, give full play to the social integration function of the community, based on geography and interest as the link, consciously set up community recreation and entertainment organizations, mutual assistance service stations, and public affairs council, attract the participation of rural migrants and residents, strengthen the communication and integration of rural migrants and urban residents. In addition, carry out innovative practices of residential models such as “selective neighborhoods” and “youth apartments” in open communities with a certain foundation and absorb rural migrants to achieve communication integration and social embedding in mixed community spaces. Additionally, efforts should be made to enhance the human capital of rural migrants, strengthening their competitiveness in the labor market.

Finally, it should be pointed out that this study still has some shortcomings. First, this study only focuses on the social integration of rural migrants at the psychological level, and social integration also includes economic integration, cultural integration, etc., and further research is needed on these issues. Second, due to data limitations, this paper mainly analyzes the pilot cities of social integration. These cities are all large, while the situation of small and medium-sized cities may differ from those of large cities, and further research is needed. Third, there are some limitations to our study, which to some extent may affect the comprehensiveness of the study due to the unavailability of up-to-date publicly available survey data on the residential choices of rural migrant communities. In future studies, we will conduct a field questionnaire survey to further strengthen our findings.

### Supplementary Information

**Supplementary Material 1.** 

## Data Availability

The datasets used in the current study is publicly available from China Migration Population Service Center [https://www.chinaldrk.org.cn].
