# Supplementary material for: How does choice of residential community affect the social integration of rural migrants: insights from China
Source: BMC Psychol. 2024 Mar 4;12:119. doi: 10.1186/s40359-024-01617-9 (PMC10910781; doi:10.1186/s40359-024-01617-9)
Supplement: Supplementary file 1 — Supplementary Material 1. [file 40359_2024_1617_MOESM1_ESM.docx]

**Appendix**

**Table A1** Determinants of residential community choice for rural migrants: Probit model results.

| **Variables** | **(1)** |
| --- | --- |
| Age | 0.0043** |
|  | (0.0019) |
| Gender | -0.0195 |
|  | (0.0259) |
| Education | 0.3231*** |
|  | (0.0282) |
| Marriage | -0.1599*** |
|  | (0.0391) |
| Health | 0.0182 |
|  | (0.0135) |
| Income | 0.0780*** |
|  | (0.0280) |
| Inter-city flows | -0.0384 |
|  | (0.0681) |
| Inter-provincial flows | -0.2033*** |
|  | (0.0740) |
| Length | 0.0034 |
|  | (0.0033) |
| Housing | 1.2260*** |
|  | (0.0479) |
| Employer | -0.0683** |
|  | (0.0304) |
| City effects | YES |
| Constant | -1.6434*** |
|  | (0.2520) |
| Pseudo R2 | 0.1072 |
| Observations | 13,134 |

Note: Robust standard errors in parentheses. *** p < 0.01, ** p < 0.05.
